# Research Progress on Nano-TiO_2_ Photocatalytic Degradation of Automobile Exhaust

**DOI:** 10.3390/molecules31091439

**Published:** 2026-04-27

**Authors:** Yang Yang, Sitong Bie, Haiping Liu, Jie Li, Xiaoxue Zhang, Zijun Zhang

**Affiliations:** College of Civil and Architectural Engineering, Heilongjiang Institute of Technology, Harbin 150050, China; biesitong@hljit.edu.cn (S.B.); liuhaiping@hljit.edu.cn (H.L.); lijie1@hljit.edu.cn (J.L.); zhangxiaoxue@hljit.edu.cn (X.Z.); zhangzijun@hljit.edu.cn (Z.Z.)

**Keywords:** nano-TiO_2_, photocatalysis, degradation, automobile exhaust, research progress

## Abstract

Nano-TiO_2_ is widely used in many industrial fields due to its unique physical and chemical properties. In recent years, it has become a core material in the research of road engineering for degrading automobile exhaust. Under ultraviolet irradiation, it can excite electron-hole pairs and use its strong redox capacity to decompose automobile exhaust and improve air quality. From the perspectives of materials, performance and engineering application, this paper briefly describes the structure and physicochemical properties of nano-TiO_2_, reviews the recent research progress of nano-TiO_2_ in the photocatalytic degradation of automobile exhaust, systematically compares the effects of various strategies such as incorporation methods and modified materials on exhaust degradation efficiency, and conducts a quantitative analysis of performance differences. It is pointed out that insufficient road durability, poor compatibility with pavement materials and limited adaptability to unconventional environments are the main current problems and challenges in this research direction. The future development directions such as developing self-healing composite systems and constructing machine learning prediction models are also prospected.

## 1. Introduction

Nano materials are ultra-fine materials with many excellent properties not found in traditional materials, such as surface effect, small size effect, enhanced specific surface area and magnetism [1]. Titanium dioxide (TiO_2_) is a white and non-toxic oxide with many good properties such as simple preparation process, environmental friendliness, photocatalysis, antibacterial property, corrosion resistance and weather resistance [2]. It is also rich in resources and low in price. Nano-TiO_2_ is widely used in catalytic materials [3], environmental protection [4,5,6], ceramic coatings [7], medical antibacterial [8], cosmetics [9], superhydrophobic materials [10] and other fields, and it is one of the most widely used nano materials at present. Since the 20th century, the transportation industry has developed rapidly and the number of motor vehicles has increased significantly. The accompanying problems have also increased. The increasing number of automobiles release more and more air pollutants, which aggravate the degree of environmental pollution. Traditional subgrade and pavement materials cannot actively reduce the environmental pollution caused by automobile exhaust. We need higher quality and more environmentally friendly pavement materials, which have important practical significance for the future technological development of road engineering and the improvement of residents’ living environment. At present, photocatalytic environmental protection pavement is regarded as one of the most promising functional facilities in road infrastructure. Therefore, the photocatalytic degradation of automobile exhaust has become a research hotspot in pavement materials in recent years, aiming to reduce the pollution level by degrading the polluting gases in motor vehicle exhaust through pavement materials. Nano-TiO_2_ can degrade some organic and inorganic pollutants in automobile exhaust through photocatalytic reaction [11], and reduce the impact of automobile exhaust on urban air and residents’ physical health.

Existing reviews mainly focus on two aspects. One is the crystal form regulation, surface modification and photocatalytic principle of nano-TiO_2_ [12], which do not analyze the performance stability combined with the complex pavement environment. The other is the application of photocatalytic technology in atmospheric governance [13], which mentions the pollutant degradation effect but does not correlate with the engineering characteristics of pavement. A small number of recent reviews related to photocatalytic pavement only focus on engineering cases [14], without systematically comparing the advantages and disadvantages of nano-TiO_2_ incorporation methods or analyzing common problems such as insufficient visible light response. Compared with existing studies, this paper focuses on the application scenario of pavement engineering and combines material characteristics with pavement service requirements. It integrates the research results of the whole process of materials, performance and application, so as to provide support for the engineering practice guidance of photocatalytic environmental protection pavement. Aiming at the current technical bottlenecks, it puts forward future directions combined with recent data to provide reference for the large-scale application of the technology.

## 2. Structure and Physicochemical Properties of Nano-TiO_2_

### 2.1. Structure of Nano-TiO_2_

**Figure 1:**
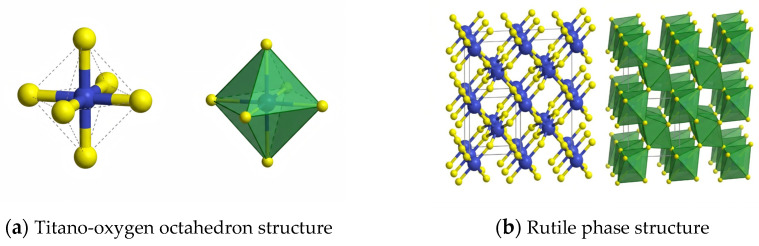

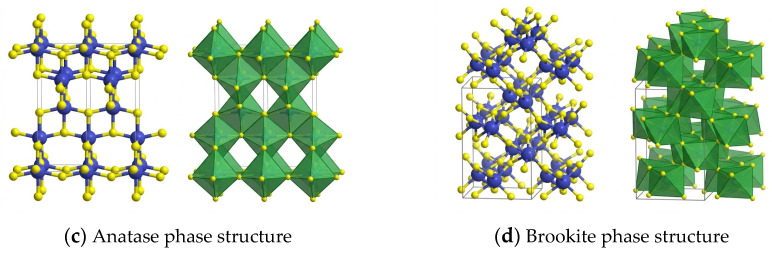
Structures of different TiO_2_ crystals [18,19,20].

Nano-TiO_2_ is a polycrystalline semiconductor compound. The common crystal forms include rutile, anatase and brookite. Their crystal structures are shown in Figure 1, which show different properties due to the differences in crystal structure [15]. Among them, rutile belongs to the tetragonal crystal system. One Ti^4+^ is at the center of the crystal lattice, and six O_2_^−^ are at the edges of the TiO_6_ octahedron. Two TiO_2_ molecules form a unit cell, which is the most stable crystal form under most conditions. The structure of anatase is similar to that of rutile, but each unit cell contains four TiO_2_ molecules, which are stable at low temperature and begin to transform into rutile when the temperature reaches 500 °C. Brookite belongs to the orthorhombic crystal system, and six TiO_2_ molecules form a unit cell. It only exists in natural minerals with a small content and directly transforms into rutile when the temperature is above 650 °C, with low application value. At present, the commonly used nano-TiO_2_ is mostly composed of 80% anatase and 20% rutile [16,17].

### 2.2. Physicochemical Properties of Nano-TiO_2_

Nano-TiO_2_ is a white amphoteric oxide in solid or powder form, with morphologies including spherical, rod-like, tubular and porous structures. These different morphologies have a significant impact on its specific surface area and the distribution of active sites, thus affecting its photocatalytic performance. The average particle sizes of anatase and rutile are 11 nm and 50 nm respectively [1]. Rutile nano-TiO_2_ has high hardness and wear resistance, so it is often used as a coating reinforcement material to improve the mechanical strength and durability of the substrate [21]. Nano-TiO_2_ nanoparticles have high specific surface area which provides abundant active sites and enhances adsorption, and reaction capacity and surface hydroxyl characteristics which are easy to bond with other molecules and improve surface activity [1]. In terms of optics, nano-TiO_2_ shows strong absorption characteristics in the ultraviolet region with a wavelength less than 387 nm, which is transparent in the visible light region [22]. In terms of chemical stability, although it has excellent acid and alkali corrosion resistance and oxidation resistance, it may still dissolve in strong acid or strong alkali environments [1], which limits its application in complex environments. At the same time, as a typical n-type semiconductor, it has high electron mobility, and the photoelectric conversion efficiency in dye-sensitized solar cells can reach more than 10% [23].

In summary, nano-TiO_2_ has a significant prospect in the development of visible light catalysis, environmental friendliness and multi-functional composite materials by virtue of its strong photocatalytic activity, chemical stability, ultraviolet shielding ability and adjustable surface properties.

## 3. Mechanism of Nano-TiO_2_ Photocatalytic Degradation of Automobile Exhaust

**Figure 2:**
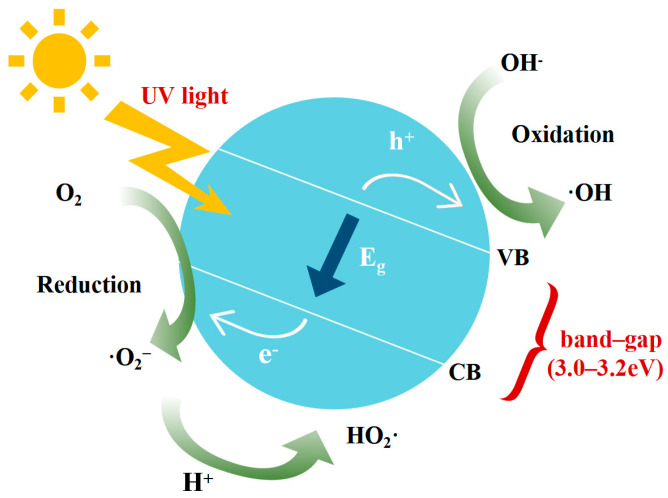
Principles of photocatalytic reaction.

TiO_2_ is a semiconductor material with photocatalytic redox capacity. The principle of nano-TiO_2_ photocatalytic reaction is as follows. Under the irradiation of light with energy higher than the band gap energy, the valence band electrons are excited to jump to the conduction band, leaving holes at the original valence band positions. The conduction band electrons jump back to the valence band and recombine with holes to release energy. The surface holes act as oxidants to oxidize hydroxyl ions into hydroxyl radicals, and electrons act as reducing agents to reduce molecular oxygen into superoxide anion radicals [24]. The above reactions are shown in Formulas (1)–(10), and the reaction process is shown in Figure 2.

(1) $${\mathrm{TiO}}_{2}+\mathrm{hv}\;\to {\;\mathrm{TiO}}_{2}{\;+\;h}^{+}+e^{-}$$

(2) $$h^{+}+e^{-}\to \mathrm{Energy}$$

(3) $${\mathrm{OH}}^{-}{+\;h}^{+}\to \cdot \;\mathrm{OH}$$

(4) $$H_{2}{O+h}^{+}\to {\;H}^{+}+\cdot \;\mathrm{OH}$$

(5) $$O_{2}+e^{-}\;\to \;\cdot {\;O}_{2}^{\;-}$$

(6) $$H_{2}O+\cdot {\;O}_{2}^{\;-}\to \;{\mathrm{HO}}_{2}\cdot +\mathrm{OH}\cdot$$

(7) $${2\mathrm{HO}}_{2}\cdot \to {\;O}_{2}{\;+\;H}_{2}O_{2}$$

(8) $${\mathrm{HO}}_{2}\cdot {+\;H}_{2}O+e^{-}\to H_{2}O_{2}+\mathrm{OH}\cdot$$

(9) $$H_{2}O_{2}{\;+\;e}^{-}\;\to \cdot \;\mathrm{OH}\;+\;{\mathrm{OH}}^{-}$$

(10) $$H_{2}O_{2\;}+\cdot O_{2}^{\;-}\;\to \;\cdot \mathrm{OH}+{\mathrm{OH}}^{-}{+O}_{2}$$

**Figure 3:**
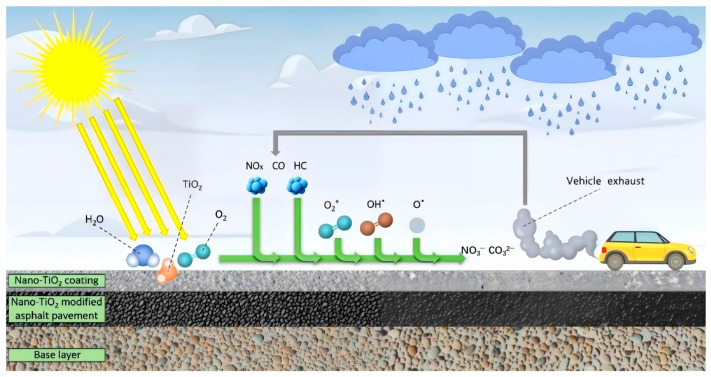
A schematic view of the photocatalytic reaction of incorporating nano-TiO_2_ into asphalt pavement [14].

In the actual road application process, under ultraviolet conditions, nano-TiO_2_ acts as a catalyst, which can degrade CO, HC, and NO_x_ in automobile exhaust into carbonate and nitrate, which can then be washed away by rain or street sprinkling to avoid biological inhalation. The degradation reaction formula is shown in Formulas (11)–(18) [25,26,27,28,29,30,31], and the reaction process is shown in Figure 3. At the macro level, nano-TiO_2_ photocatalytic environmental protection pavement has significant advantages. The large-scale and long-mileage characteristics of road construction provide a broad application scenario for the nano-TiO_2_ photocatalytic degradation of exhaust. As a close contact of vehicle exhaust, the pavement can realize real-time treatment of the exhaust. At the same time, ultraviolet irradiation is an essential condition for photocatalytic reaction, and most traffic pavements can naturally obtain sunlight, which provides a free and sustainable energy source for photocatalytic reaction [32].

CO degradation produces carbonates and CO_2_:

(11) $$\mathrm{CO}\;+\cdot \mathrm{OH}\;\to {\;\mathrm{CO}}_{2}\;+{\;H}^{+}$$

(12) $${\mathrm{CO}}_{2}+{\;H}_{2}O\;\to \;{\;\mathrm{HCO}}_{3}^{\;-}+H^{+}$$

HC (using methane CH_4_ as an example) is degraded into CO_2_ and H_2_O:

(13) $${\mathrm{CH}}_{4}\;+\cdot \mathrm{OH}\;\to \cdot {\mathrm{CH}}_{3}\;+H_{2}O$$

(14) $$\cdot {\mathrm{CH}}_{3}{+O}_{2}{+H}_{2}O\to {\;\mathrm{CO}}_{2}{+\mathrm{HO}}_{2}\cdot {+H}^{+}$$

NO_x_ degradation produces nitrate:

(15) $$\mathrm{NO}\;+\cdot \;\mathrm{OH}\;\to {\;\mathrm{HNO}}_{2}$$

(16) $${\mathrm{HNO}}_{2\;}+\cdot \;\mathrm{OH}\;\to {\;\mathrm{NO}}_{2}{\;+\;H}_{2}O$$

(17) $${\mathrm{NO}}_{2}+\cdot \;\mathrm{OH}\;\to {\mathrm{HNO}}_{3}$$

(18) $${\mathrm{HNO}}_{3}{\;+\;H}_{2}O\;\to {\mathrm{NO}}_{3}^{\;-}{\;+\;H}_{3}O^{+}$$

## 4. Research on the Application of Nano-TiO_2_ Photocatalytic Degradation of Automobile Exhaust

Although nano-TiO_2_ shows great potential in the pavement application of photocatalytic degradation of automobile exhaust, its actual popularization still faces multiple technical bottlenecks. Aiming at the existing problems, researchers have carried out research from multiple dimensions such as durability improvement, carrier structure design, the expansion of visible light response range and analysis of influencing factors, and formed a series of research results with application value.

### 4.1. Optimization Methods to Improve Durability and Engineering Adaptability

Nano-TiO_2_ is easy to lose due to abrasion, rain scouring, freeze–thaw cycles and other factors in pavement use, and the traditional incorporation method has insufficient adaptability with pavement substrates, which directly affects the long-term photocatalytic effect and road performance. Existing studies have realized the balance between the durability and engineering practicability of nano-TiO_2_ photocatalytic pavement through the combined scheme of incorporation method improvement and material modification design.

The traditional incorporation methods are mainly divided into mixing method and coating method. The mixing method mixes nano-TiO_2_ powder into asphalt mixture to replace part of mineral powder. Although it has strong wear resistance and basically does not affect the various properties of the asphalt mixture, a large amount of incorporation is needed to ensure the degradation efficiency, which not only increases the cost, but also may reduce the pavement uniformity due to particle agglomeration [33,34]. The coating method mixes nano-TiO_2_ with water or binder to prepare a catalyst solution, which is then coated on the road surface to play its role. A small amount of incorporation can achieve a high degradation rate, but the bonding force between the coating and the pavement surface is weak, and mass loss is easy to occur due to abrasion, thus affecting the exhaust degradation rate [33]. To break this limitation, it is necessary to improve and innovate on the basis of traditional methods. Adding nano-TiO_2_ to the fog seal material of asphalt pavement not only retains the advantage of the low incorporation amount of the coating method, but also improves the durability by virtue of the permeation combination between the fog seal and the pavement. The actual measurement shows that its abrasion loss rate is about 50% lower than that of water-based coating, and the impact on the pavement skid resistance is within the scope permitted by the specification [35]. However, the degradation rate of this method is slightly poor, which may be related to the fact that some TiO_2_ particles are covered by asphalt. Considering that traffic marking is an essential requirement for the normal operation of roads, taking advantage of the characteristics of marking coatings with less contact with tires and low wear rate, nano-TiO_2_ is mixed into marking coatings. When the additional amount is 4%, the NO degradation rate under natural light can reach 71%, and the degradation efficiency attenuation is small during the service cycle of the marking [36]. However, this method only acts on the marking area, cannot realize the whole area degradation of the pavement, and has limited application scenarios. In terms of material modification design, some studies have cross-linked nano-TiO_2_ on the surface of fine rubber powder to obtain a three-dimensional network for adsorbing and degrading exhaust gas as much as possible. At the same time, the chemical cross-linking reaction between nano-TiO_2_ and rubber powder makes the composite degradation particles have stronger wear resistance, compression resistance and water washing resistance. Under the same conditions, the degradation efficiency of composite degradation particles is higher than that of pure nano-TiO_2_ powder [37]. To solve the compatibility between degradation materials and pavement carriers, some studies have developed a cellulose polymer stabilizer with polyacrylamide chain grafting. This stabilizer can significantly improve the compatibility and stability between degradation materials and carriers. At the incorporation amount of 2% and storage time of 180 h, the stability coefficient is not more than 5.5%, which meets the requirements of engineering application [38,39].

In summary, the core of improving durability and adaptability is to balance the relationship among degradation efficiency, material loss and road performance. Although the existing methods have made various breakthroughs, the coordination of global performance, long-term stability and cost control still needs to be optimized, which provides a direction for the subsequent carrier structure innovation and composite modification.

### 4.2. Efficiency Improvement Strategies of Porous Carrier and Structural Design

On the basis of solving the durability problem, how to improve the degradation efficiency has become the key. The degradation efficiency of nano-TiO_2_ is directly related to its specific surface area, the number of active sites and the contact opportunity of pollutants. The traditional particles have insufficient exposure of active sites due to agglomeration. The porous carrier and special structural design can effectively solve this problem by optimizing the spatial distribution and interface characteristics of materials, and become the key path to improve the degradation efficiency.

The porous pavement structure makes nano-TiO_2_ more exposed to light and exhaust through large voids, and at the same time improves the drainage performance in rainy days and reduces the scouring loss of catalysts by rainwater. Some researchers have integrated photocatalytic materials into porous pavement to endow the pavement with additional exhaust degradation capacity [40,41], and determined the key indicators for evaluating photocatalytic performance according to experimental data [42]. Some studies have also sprayed nano-TiO_2_ on the road surface to prepare photocatalytic environmental protection pervious concrete, combining two new pavement research technologies to exert greater environmental benefits [43]. The microporous membrane embedding technology, such as preparing honeycomb-like microporous structure on the asphalt surface by breath figure method and fixing TiO_2_ particles in the holes, not only expands the sunlight contact area, but also reduces the direct effect of abrasion [44,45]. However, the pore membrane size is easily affected by the humidity of the construction environment, resulting in insufficient performance stability in batch application. High specific surface area carrier is also the core means to improve efficiency. SiO_2_ aerogel carrier is a typical representative. The TiO_2_-SiO_2_ aerogel has a specific surface area of 200 m^2^/g, with a continuous three-dimensional network and uniform pore size. The rich pore structure not only provides a large number of active sites, but also can adsorb and enrich exhaust pollutants. Its photonic efficiency of NO_x_ is higher than that of ordinary nano-TiO_2_ [46]. However, the aerogel has low mechanical strength, and it is easy to break when directly mixed into asphalt mixture, so it is recommended to be used in combination with resin for reinforcement.

On the whole, the porous carrier design needs to balance the high specific surface area and engineering strength. Although aerogel and mesoporous materials have significant advantages in specific surface area, their mechanical properties are insufficient. In the future, the coordination of structure and performance can be further optimized through carrier surface modification and in situ growth of TiO_2_.

### 4.3. Modification Methods and Efficiency Comparison for Expanding Visible Light Response

Although a variety of incorporation methods and carrier structure designs have been developed, the spectral response limitation and easy loss of nano-TiO_2_ itself limit its degradation efficiency. Traditional TiO_2_ has a large band gap and can only respond to photons in the ultraviolet region, which accounts for only about 4% of sunlight, while visible light, which accounts for about 45% of sunlight, is not effectively utilized. In addition, the recombination rate of electron-hole pairs in TiO_2_ is relatively high, leading to low quantum yield and poor degradation efficiency in photocatalytic reaction [47]. Existing studies have effectively narrowed the band gap, inhibited carrier recombination and significantly improved the catalytic performance under visible light through modification methods such as ion doping, semiconductor composite and carbon material composite [48,49]. The technical characteristics, advantages, and disadvantages of each method are shown in Table 1.

In recent years, the research on the modification of nano-TiO_2_ for visible light response has gradually shifted from early single doping to multi-component synergistic strategies such as semiconductor composite and carbon material composite, and the degradation efficiency has been improved compared with single modification. However, the current research still has the following core problems. The analysis of micro mechanisms is insufficient, focusing on the improvement of macro performance, and the explanation of key mechanisms such as lattice interaction and charge transfer path is inadequate, resulting in the difficulty of the precise regulation of modification effects. The engineering applicability is insufficient. Most laboratory studies are based on ideal conditions such as single pollutant and constant environment, which are disconnected from the actual pavement conditions such as abrasion scouring and the coexistence of multiple pollutants, and the long-term stability of materials lacks verification. The contradiction between cost and large-scale application is prominent. The cost of modifiers such as graphene and precious metals is high, and some multi-step synthesis processes are complex, which are difficult to adapt to the large-scale construction requirements of pavement engineering. In the future, research should rely on characterization technologies such as in situ XRD and XPS to establish a quantitative relationship between modification parameters, microstructures, and photocatalytic performance to consolidate the theoretical support. Develop an integrated scheme of modification and protection and verify the environmental adaptability of materials through long-term outdoor exposure tests. At the same time, use industrial wastes such as steel slag and fly ash as low-cost modifiers, optimize simplified processes such as one-step hydrothermal method, and promote the transformation of laboratory results into engineering applications.

### 4.4. Analysis of Influencing Factors of Photocatalytic Degradation Reaction

The nano-TiO_2_ photocatalytic reaction is affected by various surrounding conditions. Although extensive photocatalytic experiments have been carried out in the laboratory to verify the feasible degradation of nano-TiO_2_, in situ monitoring shows that the purification performance is highly dispersed [93]. Therefore, it is crucial to determine the influence mechanism of photocatalytic reaction from different factors before large-scale popularization. Table 2 systematically sorts out the core influencing factors of nano-TiO_2_ photocatalytic degradation efficiency and clarifies the correlation trend between each factor and efficiency.

In the actual pavement environment, the coupling effect between factors often significantly affects the degradation efficiency, among which the antagonistic effect of temperature and humidity is the most critical. The combination of the two leads to the difficulty of achieving optimal efficiency through the optimization of a single factor. The coupling of ultraviolet irradiance with temperature and humidity is more complex and there are controversial points. The root cause may lie in the difference in experimental conditions. In the laboratory environment with constant temperature and humidity, irradiation only reflects the leading role of electron-hole generation, showing a positive correlation. In outdoor experiments, the temperature and humidity fluctuations caused by irradiation cover up the direct promoting effect, leading to the decrease in efficiency under high irradiation. At the same time, the type of pollutants will also affect the results. Pollutants such as NO_x_ that compete with water for adsorption sites are more susceptible to the negative effects of dehumidification under high irradiation. On the whole, the influences of crystal structure, material physical properties, humidity, temperature, mixture form, construction process and additive content on degradation efficiency have formed a strong consensus, which can directly guide engineering practice, while the influence of ultraviolet irradiation needs to be judged dynamically in combination with the environment. Future research should focus on the construction of a quantitative model of multi-factor coupling, incorporate the interaction effects of temperature, humidity, and irradiance into the prediction system, avoid the actual performance deviation caused by single factor optimization, and provide accurate support for the parameter design of photocatalytic pavement in different climate zones.

## 5. Conclusions and Prospects

The application of nano-TiO_2_ in road engineering for degrading automobile exhaust is of great significance to environmental protection and sustainable development, and conforms to the development concept of green and environmental protection roads and low-carbon cities in the new era. Due to the late start of the research on nano-TiO_2_ photocatalytic degradation of automobile exhaust, the technical feasibility has been verified through laboratory research in this field at present. However, from the achievement transformation to the large-scale engineering application, there are still core bottlenecks, and targeted breakthrough directions need to be put forward as follows.

(1) The nano-TiO_2_ photocatalyst itself does not participate in the redox reaction and is not consumed with time, which is theoretically permanent. However, in the actual application process, it will be lost due to the influence of pavement wear and surrounding environmental factors, and there is no substrate-catalyst matching scheme that takes into account strong adhesion and catalytic activity, which is difficult to meet the pavement durability cycle. In the future, we can focus on developing adhesives with self-healing functions to extend the service life of catalysts. For example, microcapsule repair agents are compounded with nano-TiO_2_. When microcracks appear in the coating due to abrasion, the microcapsules rupture and release the repair agents to realize the secondary bonding of the interface and reduce the annual loss rate. At the same time, explore new carriers such as porous ceramics and basalt fibers, and lock nano-TiO_2_ particles through pore structure to take into account high specific surface area and wear resistance.
(2) The compatibility between nano materials and asphalt is generally poor, and nano particles have agglomeration effect. How to effectively disperse nano materials into asphalt mixture in the production process is still a great challenge, and how to maximize the role of nano-TiO_2_ in the pavement. In the future, ultrasonic assistance, silane coupling agent surface modification and other technologies can be used to reduce the particle agglomeration effect and improve the dispersion uniformity. At the same time, carry out research on the synergistic effect of multiple additives, screen anti-rutting agents, anti-aging agents and other additives compatible with nano-TiO_2_, and realize the double improvement of degradation efficiency and road performance.
(3) Most of the existing studies are based on laboratory conditions such as high concentration exhaust and constant temperature and humidity, which are far from the actual road conditions. There is also a lack of efficiency prediction models under the coupling of multiple factors such as temperature, humidity and irradiance, which cannot accurately predict the actual degradation effect. In the future, it is necessary to carry out special degradation experiments for unconventional environments such as low temperature, high humidity and high dust. At the same time, introduce machine learning methods, integrate multi-dimensional data to build a high-precision efficiency prediction model, and provide support for the parameter design of different climate zones such as the cold region in Northeast China and the high humidity region in South China.
(4) Current research focuses on exhaust degradation efficiency, but ignores the potential impacts of the whole technology chain, such as the pollution risk of nano-TiO_2_ particle shedding on soil or water bodies, and the recycling path of materials after pavement abandonment. A practical engineering technology should not only emphasize the advantages of improving the environment and prospering the economy, but also consider the relevant adverse effects from a long-term perspective. An ecological risk early warning model can be developed at the same time to ensure that the technology meets the requirements of ecological security while exerting environmental benefits, and promoting the nano-TiO_2_ photocatalytic pavement from laboratory verification to large-scale engineering application.

## Figures and Tables

**Table 1:** Modification methods for extending visible-light response of nano-TiO_2_ photocatalytic materials.

Modification Type	Typical Solution	Core Mechanism	Limitations
Nonmetallic element doping [50,51,52,53]	S, N, C, I, B, F	Nonmetallic atoms replace O atoms in the TiO_2_ lattice or dope into the lattice interstitially. This introduces new impurity levels and reduces the band gap so that the material can absorb visible light.	High concentration doping easily forms carrier recombination centers and reduces quantum efficiency. Doped elements tend to desorb at high temperatures and show poor long-term stability.
Nonmetallic material loading [54,55,56,57,58,59]	Porous nonmetallic minerals, Glass, Carbon materials, Polymer materials	A porous nonmetallic TiO_2_ composite system is formed to solve the agglomeration of nano TiO_2_ particles. Porous mineral composites have strong adsorption ability for pollutants.	Most nonmetallic supports have no catalytic activity and only play a physical supporting role. Some supports have poor light transmittance and affect light absorption efficiency.
Metal ion doping [60,61,62,63,64,65]	Fe^3+^, Mo^5+^, Os^3+^, Rh^3+^	After metal ions enter the TiO_2_ lattice, they cause lattice distortion and form local energy levels. Metal ions can also act as electron trapping centers to restrain the recombination of photogenerated electron hole pairs.	Metal ions easily undergo photochemical migration and cause agglomeration of active sites. Noble metal ions have high cost and are hard to be used on a large scale. High doping content easily leads to excessive lattice defects and reduces material stability.
Multi element co doping [66,67,68,69]	Co doping of different metal elements, Doping of metal elements with nonmetallic elements, Co doping of different nonmetallic elements	The synergistic effect among multiple dopants makes co doped TiO_2_ show higher visible light absorption than single doped TiO_2_ and effectively improves photocatalytic performance.	The type and proportion of doped elements are difficult to control accurately. Uneven doping and excessive lattice defects may become recombination centers of photogenerated carriers and reduce catalytic efficiency.
Semiconductor composite [70,71,72,73,74,75,76,77,78,79,80,81,82,83,84,85]	Traditional types (type I cross band gap, type II staggered band gap and type III broken band gap), Z scheme and S scheme	Heterojunctions are constructed to realize the separation and transfer of photogenerated carriers by using the energy level difference in different semiconductors. The light response range can also be broadened synergistically.	Poor interface compatibility of heterojunctions easily increases the resistance of interface charge transfer. The composite ratio is difficult to control and excessive compounding may lead to decreased activity.
Noble metal deposition [61,86,87,88]	Pt, Ag, Au, Pd	Electrons transfer from the TiO_2_ surface with higher energy levels to the noble metal surface with lower energy levels. When the energy levels of the two surfaces are equal, electron transfer stops and a Schottky barrier is formed. This effectively separates photogenerated electron hole pairs and improves the photocatalytic activity of TiO_2_.	The cost of noble metal deposition is very high. TiO_2_ modified by noble metal deposition has high selectivity in photocatalytic degradation of organic matters, which limits the application of these materials in pollution control to a certain extent.
Dye sensitization [89,90,91,92]	organometallic dyes containing transition metals and organic dyes composed of organic chromophores	Sensitizer dyes bind to the TiO_2_ surface through chemical or physical adsorption, which shifts the absorption wavelength of visible light to long waves. Thus the wavelength response range of TiO_2_ is expanded and solar energy utilization efficiency is improved.	Organic dye molecules degrade gradually under photocatalysis. Most sensitizers have weak absorption in the near-infrared band and easily compete with pollutants for adsorption.

**Table 2:** Main influencing factors on the photocatalytic degradation efficiency of TiO_2_ [93,94,95,96,97,98,99,100,101,102,103].

Influencing Factor	Relationship Trend with Degradation Efficiency	Core Influence Mechanism
Crystal structure	Mixed crystal (anatase: rutile = 3:1) > anatase > rutile	The anatase crystal structure is open with many active sites. The 3:1 mixed crystal form has intercrystalline synergy to accelerate the separation of electron-hole pairs.
Material physical properties	The increase in specific surface area, the decrease in particle size with the optimal range of 10 to 20 nm and the rich surface hydroxyl groups all lead to the improvement of efficiency	Specific surface area and particle size determine the number of active sites, and surface hydroxyl groups are the main source of ·OH free radicals.
Relative humidity	The efficiency decreases significantly with the increase in humidity when the humidity is higher than 40%	Under high humidity, water competes with nitrogen oxides for adsorption sites on the catalyst surface and forms a water film to prevent pollutants from contacting the active center.
Temperature	It shows a parabolic relationship, and the efficiency decreases when the temperature is lower or higher than the optimal temperature	Low temperature inhibits reaction kinetics, and high temperature destroys the adsorption balance of pollutants. Both lead to the increase in efficiency first and then decrease. The performance is more stable in warm seasons.
Ultraviolet irradiance	Positive correlation at low irradiance and negative correlation at high irradiance	At the low irradiance stage, the generation of electron-hole pairs is dominant, and the higher the irradiance, the higher the efficiency. At the high irradiance stage, irradiance causes temperature rise and humidity reduction, which in turn inhibits the generation of ·OH free radicals, forming a linear to nonlinear transformation.
Additive incorporation amount	Positive correlation when less than the optimal amount and the efficiency tends to be stable when more than the optimal amount	The number of active sites increases with the increase in incorporation amount at low amount, and the marginal benefit decreases due to particle agglomeration at high amount.
Mixture form	The efficiency of porous type is higher than that of dense type	The porous structure expands the contact area between photocatalyst and ultraviolet light and exhaust gas and promotes the diffusion of pollutants at the same time.
Carbon deposition on TiO_2_ surface	Negative correlation: the more carbon deposits, the lower the degradation efficiency	Carbon deposits formed by incomplete oxidation of HC during redox reaction cover the active sites of TiO_2_, block the contact between TiO_2_ and ultraviolet light, lead to catalyst poisoning and deactivation, and reduce long-term photocatalytic efficiency. The degree of deactivation is affected by reaction conditions and TiO_2_ material properties.
Construction process	The efficiency of dry method is significantly higher than that of wet method	The dry method can reduce the agglomeration of nano particles, improve their dispersion uniformity in the mixture and avoid the active sites being wrapped.

## Data Availability

No new data were created or analyzed in this study. Data sharing is not applicable to this article.

## Abbreviations

The following abbreviations are used in this manuscript:

XRD	X-ray Diffraction
XPS	X-ray photoelectric spectroscopy

## References

[1] Song C., Xiao L., Chen Y., Yang F., Meng H., Zhang W., Zhang Z., Wu Y. (2024). TiO_2_-based catalysts with various structures for photocatalytic application: A review. Catalysts.

[2] Li Z., Li Z., Zuo C., Fang X. (2022). Application of nanostructured TiO_2_ in UV photodetectors: A review. Adv. Mater..

[3] Mao C., Sun J., Zhang C., Gu J. (2024). Researchprogress in the application of compositephotocatalysts based on TiO_2_. Appl. Chem. Ind..

[4] Yang Y., Liu H., Qian G., Wu H., Wang G. (2019). Experimental Study on Degradation Efficiency of Automotive Exhaust by Nano-TiO_2_ Spraying on Pavement in Low Temperature Environment. For. Eng..

[5] Cheng C., Teng Y., Li K., Xue C. (2022). Research progress in enhanced wastewater treatment performanceofmodified TiO_2_ photocatalyst. New Chem. Mater..

[6] Stancu A.G., Râpă M., Popa C.L., Donțu S.I., Matei E., Covaliu-Mirelă C. (2025). Degradation of Emerging Plastic Pollutants from Aquatic Environments Using TiO_2_ and Their Composites in Visible Light Photocatalysis. Molecules.

[7] Shi P., Sun H., Yi G. (2023). Tribological behavior and mechanical properties of thermal sprayed TiO_2_-ZnO -and TiO_x_ ceramic coatings. Ceram. Int..

[8] Wang J., Liu D., Liu W., Wang L., Dong B. (2022). Research Progress on Photocatalytic Antibacterial Application of TiO_2_ Nano Materials. Chin. J. Appl. Chem..

[9] Bousiakou L.G., Dobson P.J., Jurkin T., Marić I., Aldossary O., Ivanda M. (2022). Optical, structural and semiconducting properties of Mn doped TiO_2_ nanoparticles for cosmetic applications. J. King Saud Univ.-Sci..

[10] Li H., Lin X., Wang H., Liu Y. (2021). Fabrication and Properties of Nano-TiO_2_ Superhydrophobic Coating. For. Eng..

[11] Li W., Ding H., Ji H., Dai W., Guo J., Du G. (2018). Photocatalytic Degradation of Tetracycline Hydrochloride Via a CdS-TiO_2_ Heterostructure Composite under Visible Light Irradiation. Nanomaterials.

[12] Nakata K., Fujishima A. (2012). TiO_2_ photocatalysis: Design and applications. J. Photochem. Photobiol. C Photochem. Rev..

[13] Schneider J., Matsuoka M., Takeuchi M., Zhang J., Horiuchi Y., Anpo M., Bahnemann D.W. (2014). Understanding TiO_2_ photocatalysis: Mechanisms and materials. Chem. Rev..

[14] Ayar P., Ruhi A., Baibordy A., Asadi Azadgoleh M., Mohammadi M.M., Abdipour S.V. (2024). Toward sustainable roads: A critical review on nano-TiO_2_ application in asphalt pavement. Innov. Infrastruct. Solut..

[15] Luan J., Liu X., Jiang P., Sun D., Fang L. (2023). Design and practice of comprehensive experimentfor nano-TiO_2_ photocatalysis. Exp. Technol. Manag..

[16] Liu B., Gong H., Liu R., Hu C. (2019). One-Step Synthesis of TiO_2_ -Au Composite andIts Performance for Photocatalytic Hydrogen Evolution. Chin. J. Appl. Chem..

[17] Li R., Xiao F., Amirkhanian S., You Z., Huang J. (2017). Developments of nano materials and technologies on asphalt materials—A review. Constr. Build. Mater..

[18] Noman M.T., Ashraf M.A., Ali A. (2019). Synthesis and applications of nano-TiO_2_: A review. Environ. Sci. Pollut. Res..

[19] Bourikas K., Kordulis C., Lycourghiotis A. (2014). Titanium dioxide (anatase and rutile): Surface chemistry, liquid–solid interface chemistry, and scientific synthesis of supported catalysts. Chem. Rev..

[20] Zhang H., Banfield J.F. (2014). Structural characteristics and mechanical and thermodynamic properties of nanocrystalline TiO_2_. Chem. Rev..

[21] Hsu C.Y., Mahmoud Z.H., Abdullaev S., Ali F.K., Naeem Y.A., Mizher R.M., Karim M.M., Abdulwahid A.S., Ahmadi Z., Habibzadeh S. (2024). Nano titanium oxide (nano-TiO_2_): A review of synthesis methods, properties, and applications. Case Stud. Chem. Environ. Eng..

[22] Ghamarpoor R., Fallah A., Jamshidi M. (2023). Investigating the use of titanium dioxide (TiO_2_) nanoparticles on the amount of protection against UV irradiation. Sci. Rep..

[23] Li Z., Wang S., Wu J., Zhou W. (2022). Recent progress in defective TiO_2_ photocatalysts for energy and environmental applications. Renew. Sustain. Energy Rev..

[24] Liang Y. (2020). Application of Photocatalytic Degradation of Automobile Exhaust Road Materials. Highway.

[25] Yu H., Dai W., Qian G., Gong X., Zhou D., Li X., Zhou X. (2020). The NO_x_ degradation performance of nano-TiO_2_ coating for asphalt pavement. Nanomaterials.

[26] Wang H., Jin K., Dong X., Zhan S., Liu C. (2018). Preparation technique and properties of nano-TiO_2_ photocatalytic coatings for asphalt pavement. Appl. Sci..

[27] Liu S., Shi Z. (2022). Study on catalytic decomposition of vehicle exhaust pavement materials by TiO_2_. Energy Rep..

[28] Ochiai T., Fujishima A. (2012). Photoelectrochemical properties of TiO_2_ photocatalyst and its applications for environmental purification. J. Photochem. Photobiol. C Photochem. Rev..

[29] Abdullah H., Khan M.M.R., Ong H.R., Yaakob Z. (2017). Modified TiO_2_ photocatalyst for CO_2_ photocatalytic reduction: An overview. J. CO_2_ Util..

[30] Zhao J., Sun J., Meng X., Li Z. (2022). Recent advances in vehicle exhaust treatment with photocatalytic technology. Catalysts.

[31] Poulakis E., Philippopoulos C. (2017). Photocatalytic treatment of automotive exhaust emissions. Chem. Eng. J..

[32] Li X., Wang F., You L., Wu S., Yang C., Zhang L., Barbieri D.M. (2023). A review on photocatalytic asphalt pavement designed for degradation of vehicle exhausts. Transp. Res. Part D Transp. Environ..

[33] Tan Y., Li L., Wei P., Sun Z. (2010). Application Performance Evaluation on Material of Automobile Exhaust Degradation in Asphalt Pavement. China J. Highw. Transp..

[34] Li J., Wang D., Jiao Y. (2024). Effect of different titanium dioxide addition methods on the performance of road surface sustainable degradation exhaust. Transp. World.

[35] Guo L., Xu S., Huang Y., Xu Y. (2017). Effect Evaluation of Automobile Exhaust Decomposition by Using Fog Seal Containing Nano-TiO_2_. J. Beijing Univ. Civ. Eng. Archit..

[36] Fang M., Peng L., Li Y., Cheng Y., Zhan L. (2022). Evaluation test of NO degradation by nano-TiO_2_ coatings on road pavements under natural light. Coatings.

[37] Liu W., Wang S.Y., Zhang J., Fan J. (2015). Photocatalytic degradation of vehicle exhausts on asphalt pavement by TiO_2_/rubber composite structure. Constr. Build. Mater..

[38] Li W., Peng G., Wu Y., Dong X., Xu S. (2021). Automobile Exhaust Degradable Asphalt Mixture Development and Engineering Application. Bull. Chin. Ceram. Soc..

[39] Xu S., Li S., Li S., Li Z., Li J., Niu Y. (2019). Development and Evaluation of Stabilizer for Automobile Exhaust GasDegradation Materials Application in Pavement. China Highw. J..

[40] Mousavi Rad S., Kamboozia N., Ameri M., Mirabdolazimi S.M. (2023). Feasibility of concurrent improvement of pollutants-absorption ability from surface runoff and mechanical performance of asphalt mixtures by using photocatalytic nanomodified porous asphalt. J. Mater. Civ. Eng..

[41] Yan W., Bi C., Lu C., Fu J., Zheng M., Ding Q., Liu J. (2025). Investigation on the Factors Affecting the Exhaust Degradation Performance of Porous Pavement Mixtures with Nano-TiO_2_ Photocatalysts. Materials.

[42] Jimenez-Relinque E., Rubiano F., Hingorani R., Grande M., Castillo A., Nevshupa R., Castellote M. (2020). New holistic conceptual framework for the assessment of the performance of photocatalytic pavement. Front. Chem..

[43] Luo G., Liu H., Li W., Lyu X. (2020). Automobile exhaust removal performance of pervious concrete with nano TiO_2_ under photocatalysis. Nanomaterials.

[44] Li X., Zhang L., Wang Y., Yang X., Zhao N., Zhang X., Xu J. (2011). A bottom-up approach to fabricate patterned surfaces with asymmetrical TiO_2_ microparticles trapped in the holes of honeycomblike polymer film. J. Am. Chem. Soc..

[45] Leng Z., Yu H. (2016). Novel method of coating titanium dioxide on to asphalt mixture based on the breath figure process for air-purifying purpose. J. Mater. Civ. Eng..

[46] Zhang S., Zhang Z., Pei J., Li R., Zhang J., Cai J., Cui J. (2018). A novel TiO_2_-SiO_2_ aerogel nanocomposite absorbent: Preparation, characterization and photocatalytic degradation effects on automobile exhaust. Mater. Res. Express.

[47] Wang T., Shen D., Xu T., Jiang R. (2017). Photocatalytic degradation properties of V-doped TiO_2_ to automobile exhaust. Sci. Total Environ..

[48] Reñones P., Fresno F., Oropeza F.E., Gorni G. (2022). Structural and Electronic Insight into the Effect of Indium Doping on the Photocatalytic Performance of TiO_2_ for CO_2_ Conversion. J. Mater. Chem. A.

[49] Liu B., Su S., Zhou W., Wang Y., Wei D., Yao L., Ni Y., Cao M., Hu C. (2017). Photo-Reduction Assisted Synthesis of W-Doped TiO_2_ Coupled with Au Nanoparticles for Highly Efficient Photocatalytic Hydrogen Evolution. CrystEngComm.

[50] Kovalevskiy N., Selishchev D., Svintsitskiy D., Selishcheva S., Berezin A., Kozlov D. (2020). Synergistic Effect of Polychromatic Radiation on Visible Light Activity of N-Doped TiO_2_ Photocatalyst. Catal. Commun..

[51] Zhang L., Lu Q., Shan R., Zhang F., Muhammad Y., Huang K. (2021). Photocatalytic Degradation of Vehicular Exhaust by Nitrogen-Doped Titanium Dioxide Modified Pavement Material. Transp. Res. Part D Transp. Environ..

[52] Lim C., An H.R., Lee H., Lee R., Choi Y., Park J.I., Yoon J., Lee H.U., Lee Y.S. (2022). Carbon-Titanium Dioxide Heterogeneous (Photo) Catalysts (C–TiO_2_) for Highly Efficient Visible Light Photocatalytic Application. Compos. Part B Eng..

[53] Liu D., Wang J., Zhou J., Xi Q., Li X., Nie E., Piao X., Sun Z. (2019). Fabricating I Doped TiO_2_ Photoelectrode for the Degradation of Diclofenac: Performance and Mechanism Study. Chem. Eng. J..

[54] Zhu P., Ren Z., Wang R., Duan M., Xie L., Xu J., Tian Y. (2020). Preparation and Visible Photocatalytic Dye Degradation of Mn-TiO_2_/Sepiolite Photocatalysts. Front. Mater. Sci..

[55] Malakootian M., Nasiri A., Amiri Gharaghani M. (2020). Photocatalytic Degradation of Ciprofloxacin Antibiotic by TiO_2_ Nanoparticles Immobilized on a Glass Plate. Chem. Eng. Commun..

[56] Pierpaoli M., Lewkowicz A., Rycewicz M., Szczodrowski K., Ruello M.L., Bogdanowicz R. (2020). Enhanced Photocatalytic Activity of Transparent Carbon Nanowall/TiO_2_ Heterostructures. Mater. Lett..

[57] Abreu-Jaureguí C., Andronic L., Sepúlveda-Escribano A., Silvestre-Albero J. (2024). Improved Photocatalytic Performance of TiO_2_/Carbon Photocatalysts: Role of Carbon Additive. Environ. Res..

[58] Ni Y., Yan K., Xu F., Zhong W., Zhao Q., Liu K., Yan K., Wang D. (2019). Synergistic Effect on TiO_2_ Doped Poly (Vinyl Alcohol-Co-Ethylene) Nanofibrous Film for Filtration and PhotocatalyticDegradation of Methylene Blue. Compos. Commun..

[59] Lapidus A., Korolev E., Topchiy D., Kuzmina T., Shekhovtsova S., Shestakov N. (2022). Self-Cleaning Cement-Based Building Materials. Buildings.

[60] Wang J., Wang Z., Wang W., Wang Y., Hu X., Liu J., Gong X., Miao W., Ding L., Li X. (2022). Synthesis, Modification and Application of Titanium Dioxide Nanoparticles: A Review. Nanoscale.

[61] Pawar T.J., Contreras López D., Olivares Romero J.L., Vallejo Montesinos J. (2023). Surface Modification of Titanium Dioxide. J. Mater. Sci..

[62] Choi W., Termin A., Hoffmann M.R. (2002). The Role of Metal Ion Dopants in Quantum-Sized TiO_2_: Correlation Between Photoreactivity and Charge Carrier Recombination Dynamics. J. Phys. Chem..

[63] Matias M.L., Pimentel A., Reis-Machado A.S., Rodrigues J., Deuermeier J., Fortunato E., Martins R., Nunes D. (2022). Enhanced Fe-TiO_2_ Solar Photocatalysts on Porous Platforms for Water Purification. Nanomaterials.

[64] Ding W., Yang Y., Li X., Yuan S., Shi R., Liu Z., Luo M. (2024). Single-Atom Mo Supported by TiO_2_ for Photocatalytic Nitrogen Fixation. Langmuir.

[65] Hu Z., Xu T., Liu P., Jin G. (2020). Developed photocatalytic semi-flexible pavement for automobile exhaust purification using iron-doped titanium dioxide. Constr. Build. Mater..

[66] She H., Hua R., Zhao J., Xia Y., Wang L., Huang J., Wang Q. (2024). Synergetic Regulation of Interfacial Electronic Structure of Cu, N Co-Doped Carbon Modified TiO_2_ for Efficient Photocatalytic CO_2_ Reduction. Chem. Eng. J..

[67] Singh I., Birajdar B. (2019). Effective La-Na Co-doped TiO_2_ Nano-Particles for Dye Adsorption: Synthesis, Characterization and Study on Adsorption Kinetics. Nanomaterials.

[68] Eldoma M.A., Alaswad S.O., Mahmoud M.A., Qudsieh I.Y., Hassan M., Bakather O.Y., Elawadi G.A., Abouatiaa A.F., Alomar M.S., Elhassan M.S. (2024). Enhancing Photocatalytic Performance of Co-TiO_2_ and Mo-TiO_2_-Based Catalysts Through Defect Engineering and Doping: A Study on the Degradation of Organic Pollutants under UV Light. J. Photochem. Photobiol. A Chem..

[69] Hu Q., Liu Y., Li W., Wang Y., Liao W., Zou H., Li J., Huang X. (2023). Enhanced Photocatalytic Hydrogen Evolution under Visible-Light Using C, N Co-Doped Mesoporous TiO_2_ Nanocrystals Templated by Ionic Liquids. Chem. Eng. J..

[70] Fu Y., Liu L., Han B., Wang J., Liu Y., Liu S. (2025). Researchphotocatalytic performance ofprogressnano0ntitanium dioxide modified high efficiency catalyst in degradingpollutants in water. J. Shenyang Norm. Univ. (Nat. Sci. Ed.).

[71] Low J., Yu J., Jaroniec M., Wageh S., Al-Ghamdi A.A. (2017). Heterojunction Photocatalysts. Adv. Mater..

[72] Kaur K., Badru R., Singh P.P., Kaushal S. (2020). Photodegradation of Organic Pollutants Using Heterojunctions: A Review. J. Environ. Chem. Eng..

[73] Kumar A., Khan M., He J., Lo I.M. (2020). Recent Developments and Challenges inPractical Application of Visible–Light–Driven TiO_2_–Based Heterojunctionsfor PPCP Degradation: A Critical Review. Water Res..

[74] Qi K., Imparato C., Almjasheva O., Khataee A., Zheng W. (2024). TiO_2_-Based Photocatalysts from Type-II to S-Scheme Heterojunction and Their Applications. J. Colloid Interface Sci..

[75] Ganguly P., Mathew S., Clarizia L., Akande A., Hinder S., Breen A., Pillai S.C. (2019). Theoretical and Experimental Investigation of Visible Light Responsive AgBiS_2_-TiO_2_ Heterojunctions for Enhanced Photocatalytic Applications. Appl. Catal. B Environ..

[76] Liu Z., Tian J., Zeng D., Yu C., Huang W., Yang K., Liu X., Liu H. (2019). Binary-Phase TiO_2_ Modified Bi_2_MoO_6_ Crystal for Effective Removal of Antibiotics under Visible Light Illumination. Mater. Res. Bull..

[77] Yu J., Wang S., Low J., Xiao W. (2013). Enhanced Photocatalytic Performance of Direct Z-Scheme gC_3_N_4_-TiO_2_ Photocatalysts for the Decomposition of Formaldehyde in Air. Phys. Chem. Chem. Phys..

[78] Hao J., Zhang S., Ren F., Wang Z., Lei J., Wang X., Cheng T., Li L. (2017). Synthesis of TiO_2_@ g-C_3_N_4_ Core-Shell Nanorod Arrays with Z-Scheme Enhanced Photocatalytic Activity under Visible Light. J. Colloid Interface Sci..

[79] Wang F., Wu Y., Wang Y., Li J., Jin X., Zhang Q., Li R., Yan S., Liu H., Feng Y. (2019). Construction of Novel Z-Scheme Nitrogen-Doped Carbon Dots/{001} TiO_2_ Nanosheet Photocatalysts for Broad-Spectrum-Driven Diclofenac Degradation: Mechanism Insight, Products and Effects of Natural Water Matrices. Chem. Eng. J..

[80] Wang W., Mei S., Jiang H., Wang L., Tang H., Liu Q. (2023). Recent Advances in TiO_2_-Based S-Scheme Heterojunction Photocatalysts. Chin. J. Catal..

[81] Su H., Wang W., Jiang H., Sun L., Kong T., Lu Z., Tang H., Wang L., Liu Q. (2022). Boosting Interfacial Charge Transfer with a Giant Internal Electric Field in a TiO_2_ Hollow-Sphere-Based S-Scheme Heterojunction for Efficient CO_2_ Photoreduction. Inorg. Chem..

[82] Cui H., Cao J., Zhao Y., Wang J., Li S., Ge K., Chen J., Yang Y. (2024). Construction of IO-B-TiO_2_/In_2_O_3_ S-SchemeHeterojunction with Photothermal Effects and its Highly Efficient Photocatalytic Reduction of CO_2_ under Full-Spectrum Light. Chem. Eng. J..

[83] Zhang P., Qin T., Li D., Wu X., Ma Y., Guo H., Xiong J., Liu X., Zhao Z., Chen L. (2025). Temperature-induced evolution of CuO_x_ clusters in CuO_x_/TiO_2_ catalyst for boosting auto-exhaust oxidation. Appl. Catal. B Environ. Energy.

[84] Wu J., Luo Y., Qin Z. (2023). Composite-modified nano-TiO_2_ for the degradation of automobile exhaust in tunnels. Constr. Build. Mater..

[85] Knapik M., Zając W., Wojteczko A., Trenczek-Zając A. (2026). Loading-Controlled Photoactivity in TiO_2_@ BiVO_4_ Heterostructures. Molecules.

[86] Huang H., Liu S., Liang B., Liang J., Lin J. (2021). Research on rapid treatment of low mass concentration ammonia nitrogenwastewater with Ag doped TiO_2_. Mod. Salt Chem. Ind..

[87] Ji L., Qin X., Zheng J., Zhou S., Xu T., Shi G. (2020). Synthesis of Ag–Carbon–TiO_2_ Composite Tubes and their Antibacterial and Organic Degradation Properties. J. Sol-Gel Sci. Technol..

[88] Shan R., Lu L., Gu J., Zhang Y., Yuan H., Chen Y., Luo B. (2020). Photocatalytic Degradation of Methyl Orange by Ag/TiO_2_/Biochar Composite Catalysts in Aqueous Solutions. Mater. Sci. Semicond. Process..

[89] Ding Z., Tian L., Yang Q., Lu P., Li D., Li L., Yang N., Xie G., Hou Y. (2023). Research progress of TiO_2_-based photocatalysts for hydrogen evolution reaction. China Nonferrous Metall..

[90] Huang L., Ai Z., Fan Y., Li P. (2023). Photodegradation of Glyphosate on Carbon Quantum Dot SensitizedTitanium Dioxide Nanotube Arrays. Guangzhou Chem. Ind..

[91] Murcia J.J., Ávila-Martínez E.G., Rojas H., Cubillos J., Ivanova S., Penkova A., Laguna O.H. (2019). Powder and NanotubesTitania Modified by Dye Sensitization as Photocatalysts for the OrganicPollutants Elimination. Nanomaterials.

[92] Guo G., Guo H., Wang F., France L.J., Yang W., Mei Z., Yu Y. (2020). Dye-Sensitized TiO_2_@ SBA-15 Composites: Preparation and their Application in Photocatalytic Desulfurization. Green Energy Environ..

[93] Cordero J.M., Hingorani R., Jiménez-Relinque E., Grande M., Borge R., Narros A., Castellote M. (2020). NO_x_ removal efficiency of urban photocatalytic pavements at pilot scale. Sci. Total Environ..

[94] Zhang W., Zhang Y.X., Jia Z., Wang F., Ding L. (2019). Test method and material design of asphalt mixture with the function of photocatalytic decomposition of automobile exhaust. Constr. Build. Mater..

[95] Hassan M., Mohammad L.N., Dylla H., Cooper S.B., Mokhtar A., Asadi S. A breakthrough concept in the preparation of highly-sustainable photocatalytic warm asphalt mixtures. Proceedings of the NSF Engineering Research and Innovation Conference.

[96] Xiao Q., Yang Y., Zhao J. (2018). Effect of Diffreent Adding Methods of Nano-TiO_2_ on Photocatalytic Degradation of Automobile Exhaust on Asphalt Mixture. Testing and Characterization of Asphalt Materials and Pavement Structures: Proceedings of the 5th GeoChina International Conference 2018–Civil Infrastructures Confronting Severe Weathers and Climate Changes: From Failure to Sustainability, Hangzhou, China, 23–25 July 2018.

[97] Xie X., Hao C., Huang Y., Huang Z. (2020). Influence of TiO_2_-based photocatalytic coating road on traffic-related NO_x_ pollutants in urban street canyon by CFD modeling. Sci. Total Environ..

[98] Kuang Y., Ding F., Peng Z., Fan F., Zhang Z., Ji X. (2023). Photocatalytic Degradation of Vehicle Exhaust by Nano-TiO_2_ Cement Slurry: Experimental Factors and Field Application. Catalysts.

[99] Chen M., Liu Y. (2010). NO_x_ removal from vehicle emissions by functionality surface of asphalt road. J. Hazard. Mater..

[100] Yasmina M., Mourad K., Mohammed S.H., Khaoula C. (2014). Treatment heterogeneous photocatalysis; factors influencing the photocatalytic degradation by TiO_2_. Energy Procedia.

[101] He K., Chen Y., Mei M. (2020). Study on influencing factors of photocatalytic performance of CdS/TiO_2_ nanocomposite concrete. Nanotechnol. Rev..

[102] Pinho L., Mosquera M.J. (2013). Photocatalytic activity of TiO_2_–SiO_2_ nanocomposites applied to buildings: Influence of particle size and loading. Appl. Catal. B Environ..

[103] Surówka M., Kobielusz M., Trochowski M., Buchalska M., Kruczała K., Broś P., Macyk W. (2019). Iron and other metal species as phase-composition controllers influencing the photocatalytic activity of TiO_2_ materials. Appl. Catal. B Environ..

